# Mammalian prion propagation in PrP transgenic *Drosophila*

**DOI:** 10.1093/brain/awy183

**Published:** 2018-07-08

**Authors:** Alana M Thackray, Olivier Andréoletti, Raymond Bujdoso

**Affiliations:** 1 University of Cambridge, Department of Veterinary Medicine, Madingley Road, Cambridge, CB3 OES, UK; 2 UMR INRA ENVT 1225 -Hôtes-Agents Pathogènes, Ecole Nationale Vétérinaire de Toulouse, 23 Chemin des Capelles, Toulouse, France

**Keywords:** PrP^Sc^, prion, infectivity, *Drosophila*, strains

## Abstract

Mammalian prions propagate by template-directed misfolding and aggregation of normal cellular prion related protein PrP^C^ as it converts into disease-associated conformers collectively referred to as PrP^Sc^. Mammalian species may be permissive for prion disease because these hosts have co-evolved specific co-factors that assist PrP^C^ conformational change and prion propagation. We have tested this hypothesis by examining whether faithful prion propagation occurs in the normally PrP^C^-null invertebrate host *Drosophila melanogaster.* Ovine PrP transgenic *Drosophila* exposed at the larval stage to ovine scrapie showed a progressive accumulation of transmissible prions in adult flies. Strikingly, the biological properties of distinct ovine prion strains were maintained during their propagation in *Drosophila.* Our observations show that the co-factors necessary for strain-specific prion propagation are not unique to mammalian species. Our studies establish *Drosophila* as a novel host for the study of transmissible mammalian prions.

## Teaser

Thackray *et al.* show authentic propagation of mammalian prions in *Drosophila melanogaster* rendered transgenic solely for PrP. The molecular and cellular co-factors necessary for strain-specific prion propagation are not therefore unique to mammals, suggesting that *Drosophila* may also be a suitable host for studying prion transmission.

## Introduction

Mammalian prions cause fatal neurodegenerative diseases such as Creutzfeldt-Jakob disease (CJD) in humans, bovine spongiform encephalopathy (BSE) in cattle and scrapie disease of sheep (Prusiner, 2004). These conditions are transmissible between individuals of the same or different species and as a consequence, animal prion diseases pose a realistic threat to human health through their zoonotic potential (Bruce *et al.*, 1997). Prions lack a conventional nucleic acid-based genome. The ‘protein only’ concept that relates to prion diseases predicts that infectious prion particles consist of PrP^Sc^ in the form of aggregates of misfolded conformers of the normal host protein PrP^C^ (Prusiner, 1982). Prion propagation occurs by template-directed nucleation whereby a prion seed induces conformational change within PrP^C^ and promotes the incorporation of nascent misfolded prion protein into growing PrP^Sc^ assemblies, which subsequently undergo fragmentation (Collinge, 2016).

PrP^C^ is a cell-surface glycoprotein attached to the membrane by a glycosylphosphatidyl inositol (GPI) anchor (Stahl *et al.*, 1987). It is now well established that PrP^C^ expression is obligatory for prion replication and prion-induced neurotoxicity (Bueler *et al.*, 1993; Mallucci *et al.*, 2003). A remarkable feature of mammalian prions is the existence of different strains of the transmissible agent that directly influence host range and the clinico-pathological features of prion disease in the affected host (Prusiner, 2004). According to the ‘protein only’ concept (Prusiner, 1982), prion strain-specific properties are dictated by the conformational arrangement of PrP^Sc^ (Bessen and Marsh, 1994; Telling *et al.*, 1996; Safar *et al.*, 1998). Changes in the replication environment of prions can induce mutational change in their strain properties (Collinge and Clarke, 2007), an event that may occur during inter-species prion transmission where PrP primary structures differ (Kimberlin *et al.*, 1987; Bartz *et al.*, 2000).

Several fundamental features of mammalian prion biology remain unresolved despite considerable efforts aimed at their elucidation (Collinge, 2016). These include lack of a full description of the molecular and cellular machinery necessary to generate infectious neurotoxic prions (Supattapone, 2014). The possibility exists that mammalian hosts, in which PrP^C^ is ubiquitously expressed and well conserved, are permissive for prion formation because they have co-evolved specific co-factors that assist in prion protein misfolding and propagation. Here we have tested this hypothesis by examining whether authentic prion propagation occurs in *Drosophila melanogaster*, an invertebrate species that is phylogenetically separated from mammals by millions of years of divergent evolution. *Drosophila* and other members of the arthropod phylum do not show the presence of an orthologous PrP gene (*PRNP*) in their genome and therefore do not normally express PrP^C^ (Rivera-Milla *et al.*, 2006).

In this study, we tested the capacity of mammalian prions to faithfully propagate in a strain-specific manner in *Drosophil*a genetically engineered to express PrP^C^ (Thackray *et al.*, 2012*b*, 2014). Exposure of larval stage PrP transgenic *Drosophila* to mammalian prions resulted in a progressive accumulation of *bona fide* prions in adult flies. Strikingly, the biological properties of the original prion strain used as inoculum were maintained during their passage in the insect host. These observations show for the first time that the molecular and cellular co-factors necessary for strain-specific prion propagation are conserved between phylogenetically diverse species and are not unique to mammalian hosts. Our studies provide a fundamental advance in the understanding of mammalian prion biology and also establish *Drosophila* as a novel methodology for the study of transmissible mammalian prions.

## Materials and methods

### Fly stocks

The *UAS*-PrP fly lines w; M{VRQ-PrP(GPI), 3xP3-RFP.attP}ZH-51D and w; M{ARQ-PrP(GPI), 3xP3-RFP.attP}ZH-51D transgenic for ovine V^136^R^154^Q^171^ (VRQ) or A^136^R^154^Q^171^ (ARQ) PrP, respectively, expressed with an N-terminal leader peptide and C-terminal GPI signal sequence, were generated by PhiC31 site-specific transformation by BestGene as previously described (Thackray *et al.*, 2012*c*). The following fly lines were obtained from the Department of Genetics, University of Cambridge, UK:

$$\begin{array}{l} Actin‐5C‐GAL4\left(\text{y w};\text{P}\left\{\text{w}\left[+\text{mC}\right]=\text{Act5C}‐\text{Gal4}\right\}\text{25F}0\text{1}/\text{CyO},\text{y}\left[+\right]\right)\\ Elav‐GAL4\left(\text{P}\left\{\text{w}\left[+\text{mW}.\text{hs}\right]=\text{GawB}\right\}\text{elav}\left[\text{C155}\right]\right)\\ \text{51D}\left(\text{w};\text{M}\left\{\text{3xP3}‐\text{RFP}.\text{attP}\right\}\text{ZH}‐\text{51D}\right) \end{array}$$

*Cre*-mediated removal of the red fluorescent protein (RFP) gene from the VRQ and 51D fly genome was performed by conventional fly crosses (Thackray *et al.*, 2014). PrP transgenic *Drosophila* were crossed with either the *Elav-GAL4* or *Actin-5C-GAL4* driver fly lines to derive transgenic flies that expressed PrP pan neuronally or ubiquitously, respectively*.* 51D *Drosophila* crossed with either driver fly line were used as control flies where appropriate. All fly lines were raised on standard cornmeal media at 25°C and maintained at low to medium density, and pre-mated before experimental use.

### Prion inoculation of *Drosophila*

#### Primary transmission of sheep scrapie: sheep-to-fly

*Drosophila* at the larval stage of development were exposed to brain homogenate of cerebral cortex tissue from a confirmed PG127 (alternatively referred to as DAW or G_338_) scrapie-positive sheep (SE1848/0005) (Thackray *et al.*, 2008). New Zealand-derived VRQ/VRQ scrapie-free brain tissue was used as control material. Two hundred and fifty microlitres of a 1% (w/v) sheep brain homogenate prepared in phosphate-buffered saline (PBS) pH 7.4, were added to the top of the cornmeal that contained third instar *Drosophila* larvae in 3-inch plastic vials. Following eclosion (i.e. hatching) flies were transferred to fresh non-treated vials.

#### Secondary transmission of sheep scrapie: fly-to-fly

*Drosophila* head homogenates were prepared from 30-day-old flies that had been exposed at the larval stage to scrapie-positive or scrapie-negative sheep brain material. Two hundred and fifty microlitres of a 10^−1^ (v/v) dilution of the original fly brain homogenate were added to the top of the cornmeal that contained third instar *Drosophila* larvae in 3-inch plastic vials. Flies were transferred to fresh, non-treated vials following eclosion.

#### Transmission of defined ovine prion strains in PrP transgenic *Drosophila*

*Drosophila* at the larval stage of development were exposed to 1% (w/v) mouse brain homogenate infected with a defined ovine prion strain or prion-free mouse brain homogenate as control. The different mouse-adapted prion strains used here were isolated by serial passage of sheep scrapie isolates in ovine PrP transgenic mice as previously described (Thackray *et al.*, 2011, 2012*a*).

### Preparation of *Drosophila* head homogenate

Whole flies in an Eppendorf tube were frozen in liquid nitrogen for 10 min and then vortexed for 2 min to cause decapitation. Individual fly heads were isolated and placed in clean Eppendorf tubes using a fine paint brush. PBS pH 7.4 was added to give 1 µl/head and homogenates were prepared by manual grinding of the fly heads with sterilized plastic pestles. For western blot analysis, fly head homogenate was mixed with an equal volume of 20% scrapie-free sheep brain homogenate prior to extraction and proteinase K (PK) digestion as previously described (Lacroux *et al.*, 2012) using monoclonal antibody Sha31 (Feraudet *et al.*, 2005).

### Protein misfolding cyclic amplification

Protein misfolding cyclic amplification (PMCA) was carried out as previously described (Lacroux *et al.*, 2012). The substrate consisted of 10% (w/v) ovine VRQ PrP transgenic mouse (tg338) brain homogenate in PBS pH 7.4, 0.1% Triton™ X-100 and 150 mM NaCl buffer. Five microlitres of fly head homogenate were mixed with 45 µl of substrate in 0.2 ml thin-wall PCR tubes. Sealed tubes were then placed in the horn of a Misonix 4000 sonicator for one round of 96 cycles. Each cycle consisted of a 10-s sonication step (70% of power) followed by a 14 min and 50 s incubation step. Twenty microlitres of each reaction mix were subsequently treated with PK (4 µg of PK per mg of protein) at 37°C for 2 h and the reaction stopped by adding Pefabloc® (4 mM final concentration). PK-resistant PrP was detected by western blot as previously described (Lacroux *et al.*, 2012) using monoclonal antibody Sha31 (Feraudet *et al.*, 2005).

### Mouse prion bioassay

Mouse prion bioassays were carried out in tg338 mice, which are transgenic for ovine VRQ PrP and are highly efficient for the detection of sheep scrapie infectivity (Le Dur *et al.*, 2005). Mice (*n* = 6 per inocula) were injected intracerebrally with 20 µl of diluted fly head homogenate (to give approximately two fly-head equivalents per mouse) and monitored daily until the occurrence of clinical signs of mouse prion disease. Inoculated mice were euthanized when they started to show locomotor disorders and any impairment in their capacity to feed, or at a predefined end-point for the assay (either >250 days or in some cases >670 days post-inoculation) (Andreoletti *et al.*, 2011). Brain tissue (cerebral cortex) was collected from euthanized mice and frozen for PrP^Sc^ analysis by western blot (TeSeE, Bio-Rad) or paraffin-embedded tissue (PET) blot analysis (Andreoletti *et al.*, 2011).

### Negative geotaxis climbing assay

The locomotor ability of flies was assessed in a negative geotaxis climbing assay initiated with 45 (3 × *n* = 15) age-matched, pre-mated female flies in each treatment group. *Drosophila* were placed in adapted plastic 25 ml pipettes that were used as vertical climbing columns and allowed to acclimatize for 30 min prior to assessment of their locomotor ability. Flies were tapped to the bottom of the pipette (using the same number and intensity of taps on each occasion) and then allowed to climb for 45 s. At the end of the climbing period the number of flies above the 25 ml mark, the number below the 2 ml mark and the number in-between the 2 ml and 25 ml mark was recorded. This procedure was performed three times at each time point. The performance index (PI) was calculated for each group of 15 flies (average of three trials) using the formula:

PI = 0.5 × (*n*total + *n*top − *n*bottom) / *n*total where *n*total is the total number of flies, *n*top is the total number of flies at the top, and *n*bottom is the total number of flies at the bottom. A PI value of 1 is recorded if all flies climb to the top of the tube whereas the value is 0 if no flies climb the tube past the 2 ml mark. The mean PI ± SD (standard deviation) at individual time points for each treatment group was plotted as a regression line.

### Statistical analysis

Statistical analysis of the negative geotaxis climbing assay data was performed by the paired Student *t*-test, using Prism (GraphPad Software Inc, San Diego, USA).

### Data availability

The authors confirm that the data supporting the findings of this study are available within the article and its supplementary material.

## Results

### Mammalian prions replicate in PrP transgenic *Drosophila*

**Figure 1:**
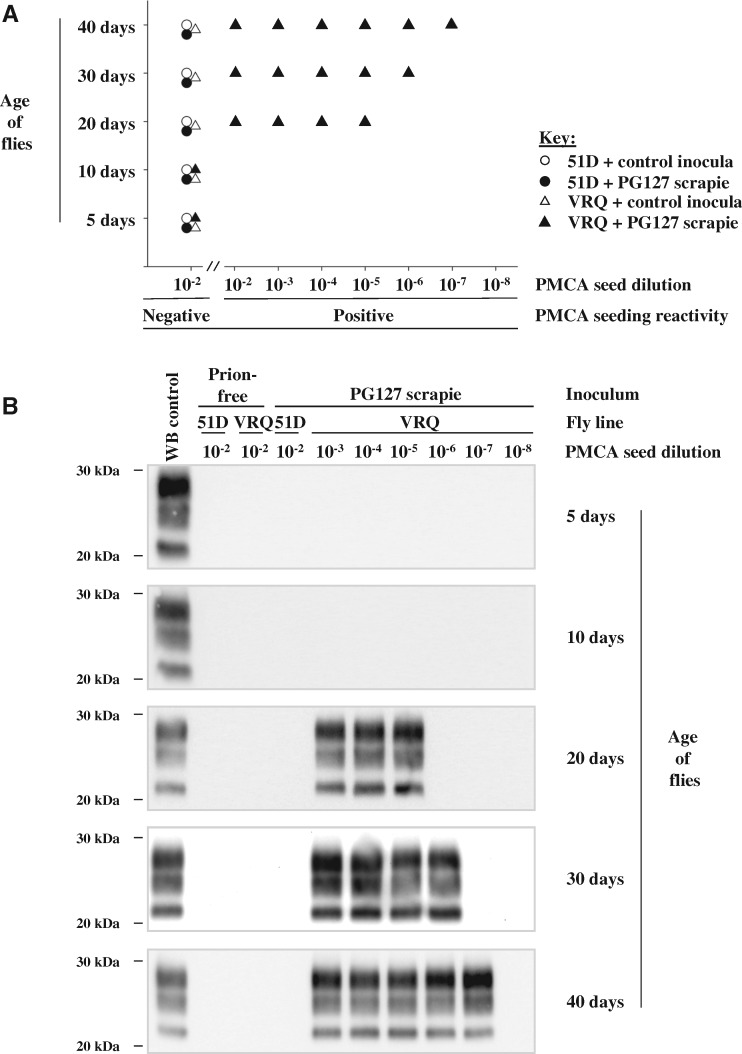
**Prion seeding activity in scrapie-exposed PrP transgenic *Drosophila.*** *Elav* × VRQ(GPI) PrP transgenic (VRQ) and *Elav* × 51D (51D) *Drosophila* were exposed at the larval stage to PG127 scrapie-infected or prion-free control sheep brain material. At various times after hatching, head homogenate was prepared from harvested flies and used as seed in PMCA reactions. (**A**) End-point titration of PMCA prion seeding activity in head homogenate from control or PG127-exposed *Drosophila.* (**B**) Western blot detection of PK-resistant PrP27–30 in PMCA reaction products seeded with control or PG127-exposed *Drosophila* head homogenate. Molecular mass markers in kDa shown on the *left*.

Transgenic *Drosophila* that express membrane-bound VRQ ovine PrP (VRQ *Drosophila* fly line) and control non-transgenic flies (51D fly line) were exposed at the larval stage to brain material prepared from either PG127 scrapie-infected or healthy prion-free sheep (Thackray *et al.*, 2012*c*, 2014). After hatching, *Drosophila* were transferred to prion-free culture tubes. At various time points (≤40 days) during their adult lifespan, groups of *Drosophila* were euthanized, decapitated and homogenate prepared from the isolated fly heads. These homogenates were used to seed *in vitro* PMCA reactions in order to reveal the presence of prion seeding activity as shown by the data in Fig. 1. PMCA mimics prion replication *in vivo*, but in an accelerated form, allowing amplification of minute quantities of PrP^Sc^ and prion infectivity (Saborio *et al.*, 2001). In PMCA reactions, a PrP^C^-containing substrate (in this study brain homogenate prepared from ovine PrP transgenic mice) is combined with the seed (in this study *Drosophila* head homogenate) that may contain minute amounts of PrP^Sc^. Following repeated cycles of incubation and sonication, the amount of PrP^Sc^ seed increases to levels at which it can be detected by conventional biochemical means, such as western blot detection of PK-resistant PrP^Sc^ as was used here.

No prion seeding activity was detected in PMCA reactions with seed prepared from mock-infected VRQ *Drosophila* head homogenate, or from scrapie-exposed or mock-infected 51D control flies (Fig. 1A). Similarly, no prion seeding activity was detected in head homogenates prepared from 5- or 10-day-old scrapie-exposed VRQ *Drosophila.* Strikingly, prion seeding activity was detected in head homogenate prepared from scrapie-infected VRQ *Drosophila* aged ≥20 days. An end-point titration of prion seeding activity, carried out using serial 10-fold dilutions of original fly head homogenate, indicated a progressive increase of prion seeding titre in the heads of prion-exposed VRQ *Drosophila* from 20 to 40 days of age (Fig. 1B). The seeding activity in 40-day-old prion-exposed VRQ *Drosophila* was 10^−4^ lower than that seen in a terminal disease-PG127 sheep scrapie inoculum (Thackray *et al.*, 2016).

**Figure 2:**
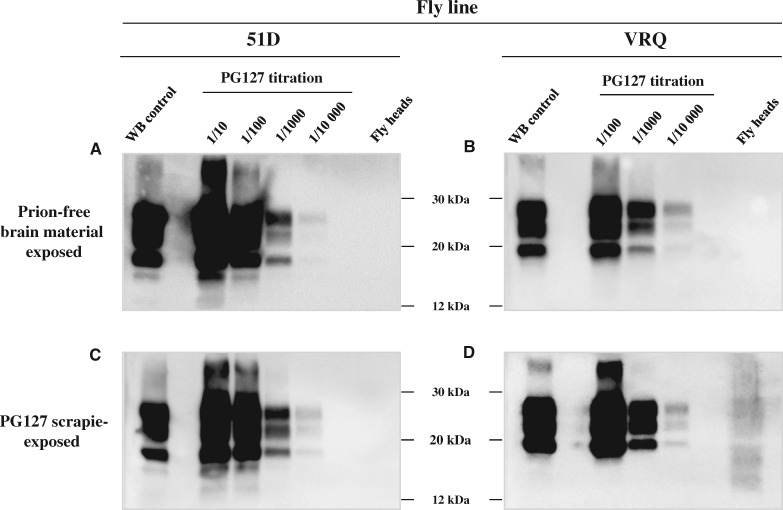
**PK-resistant PrP in scrapie-exposed PrP transgenic *Drosophila.*** *Elav* × VRQ(GPI) PrP transgenic (VRQ) and *Elav* × 51D (51D) *Drosophila* were exposed at the larval stage to PG127 scrapie-infected or prion-free control sheep brain material. At 30 days post-hatching, head homogenate was prepared from harvested flies, treated with PK and analysed by western blot to detect PK-resistant PrP. (**A** and **B**) *Drosophila* exposed to control brain homogenate. (**C** and **D**) *Drosophila* exposed to PG127 scrapie-infected sheep brain homogenate. (**A** and **C**) *Elav* × 51D *Drosophila.* (**B** and **D**) *Elav* × VRQ(GPI) PrP transgenic *Drosophila.* A titration of original PG127 scrapie-infected sheep brain homogenate is included on each blot for comparative purposes. Molecular mass markers in kDa shown in the *centre*.

We determined if the presence of the prion seeding activity in scrapie-inoculated VRQ *Drosophila* was associated with the presence of PK-resistant PrP^Sc^, a pathognomonic marker of prion disease in mammalian hosts (Prusiner, 2004). Head homogenate from 40-day-old *Drosophila* was PK-digested and analysed directly by SDS-PAGE and western blot probed with an anti-PrP monoclonal antibody. The data in Fig. 2 show that no PK-resistant PrP^Sc^ signal was detected in *Drosophila* exposed to scrapie-free sheep brain material (Fig. 2A and B), or from scrapie-treated 51D flies (Fig. 2C). Notably, a PK-resistant PrP signal was detected in head homogenate from scrapie-exposed VRQ *Drosophila* (Fig. 2D). The molecular profile of the PK-resistant PrP in VRQ *Drosophila* was clearly different to that present in the original PG127 inoculum, an expected feature considering differences in *N*-linked glycosylation that exist between *Drosophila* and mammalian hosts (Choi *et al.*, 2010; Thackray *et al.*, 2012*c*). The intensity of the PK-resistant PrP signal in the VRQ *Drosophila* head homogenate was similar to that obtained in a 10^−4^ dilution of a terminal disease-PG127 sheep scrapie inoculum. This result was consistent with the data obtained in the PMCA endpoint titration where 40-day-old VRQ *Drosophila* displayed a 10^−4^ lower seeding activity than a terminal disease-PG127 scrapie inoculum.

### Fly-to-mouse prion transmission

**Figure 3:**
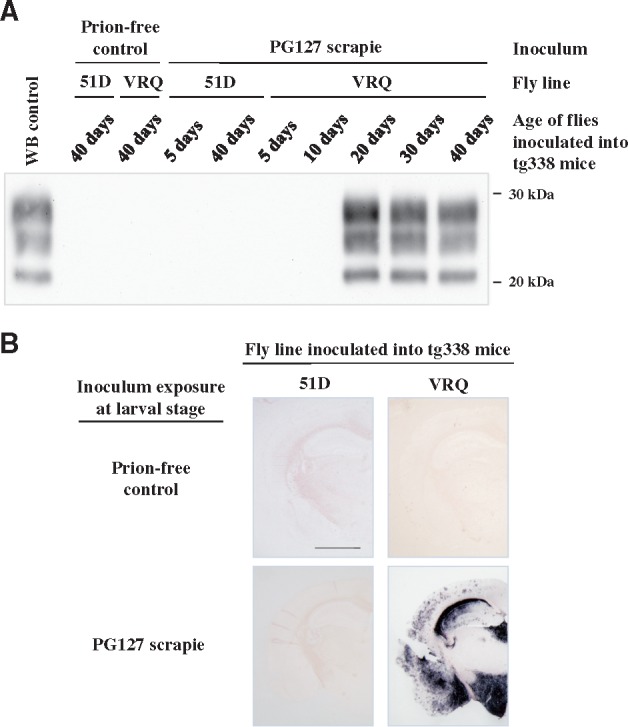
**Detection of prion infectivity in scrapie-exposed PrP transgenic *Drosophila.*** *Elav* × VRQ(GPI) PrP transgenic (VRQ) and *Elav* × 51D (51D) *Drosophila* were exposed at the larval stage to PG127 scrapie-infected or prion-free control sheep brain material. At various times after hatching, head homogenate was prepared from harvested flies and inoculated into tg338 mice. Inoculated mice were euthanized when they showed clinical signs of prion infection or after 250 days for those that did not develop clinical disease. Mice were considered positive for prion disease when PK-resistant PrP27–30 was detected in brain tissue by western blot. (**A**) Western blot detection of PK-resistant PrP27–30 in the brains of tg338 mice with clinical prion disease. Molecular mass markers in kDa shown on the *right.* (**B**) PET blot analysis of tg338 mouse brains from animals inoculated with 40-day-old *Elav* × VRQ(GPI) PrP transgenic (VRQ) or *Elav* × 51D (51D) *Drosophila* exposed to PG127 scrapie-infected or prion-free control sheep brain material. Scale bar = 150 µm.

To unequivocally demonstrate that authentic prions replicated in scrapie-exposed VRQ *Drosophila* we performed fly-to-mouse transmission. Head homogenate from flies used as a seed in the PMCA experiment shown above was inoculated into ovine VRQ PrP transgenic (tg338) mice. The transmission data are shown in Table 1. No evidence of clinical prion disease or abnormal PrP accumulation were observed in mice inoculated with head homogenates prepared from VRQ *Drosophila* and 51D flies exposed to control sheep brain homogenate. Similarly, no prion infectivity was detected in tg338 mice inoculated with head homogenates prepared from 5- or 10-day-old PG127-exposed VRQ *Drosophila.* An incomplete attack rate for clinical prion disease transmission was observed in tg338 mice inoculated with head homogenate from 20-day-old scrapie-infected VRQ *Drosophila.* Mice inoculated with head homogenates prepared from 30- and 40-day-old scrapie-exposed VRQ *Drosophila* developed a 100% attack rate for clinical prion disease transmission. The brains of tg338 mice inoculated with fly-head homogenate were probed for disease-associated PrP as shown in Fig. 3. Prion disease in mice that showed clinical signs of the condition was confirmed by western blot detection of PK-resistant PrP^Sc^ (Fig. 3A). PET blot analysis of the brains of clinically affected mice showed the typical distribution of PrP^Sc^ in PG127 scrapie-inoculated tg338 mice (Fig. 3B). No clinical signs of mouse prion disease or abnormal PrP accumulation were observed in tg338 mice inoculated with scrapie-exposed 51D fly head homogenate. This result excludes the suggestion that carry-over of the original sheep inoculum was an explanation for prion infectivity detection in scrapie-exposed VRQ *Drosophila.*

**Table 1:** Prion infectivity accumulates in scrapie-exposed PrP transgenic *Drosophila*

Fly line	Inoculum	Age of *Drosophila* inoculated into tg338 mice									
		5 days		10 days		20 days		30 days		40 days	
		Attack Rate	IP	Attack Rate	IP	Attack Rate	IP	Attack Rate	IP	Attack Rate	IP
51D	Control	0/6	>250	0/6	>250	0/6	>250	0/6	>250	0/6	>250
	PG127	0/6	>250	0/6	>250	0/6	>250	0/6	>250	0/6	>250
VRQ	Control	0/6	>250	0/6	>250	0/6	>250	0/6	>250	0/6	>250
	PG127	0/6	>250	0/6	>250	4/6	103 ± 11	6/6	89 ± 3	6/6	89 ± 2

*Elav* × VRQ(GPI) PrP transgenic (VRQ) and *Elav* × 51D (51D) *Drosophila* were exposed at the larval stage to PG127 scrapie-infected or prion-free control sheep brain material. At various times after hatching, head homogenate was prepared from harvested flies and intracerebrally inoculated into ovine PrP transgenic (tg338) mice. Inoculated mice were euthanized when they showed clinical signs of prion infection or after 250 days for those that did not develop clinical disease. Mice were considered positive for prion disease when PK-resistant PrP27–30 was detected in brain tissue by western blot. The attack rate (number of prion positive mice/total number of mice inoculated) is reported for each treatment group. The incubation period (IP) for inoculated mice, which represents the average time from inoculation to euthanasia for each inoculated group of animals, is reported in days ± SD.

To further increase the robustness of this observation, the fly-to-mouse transmission experiment was repeated twice using samples obtained in two independent experiments (using the same fly lines and the same sheep scrapie prion strain). The observed incubation times and attack rates in these repeat experiments were consistent to those recorded in the original experiment, as shown in the Supplementary material, S1. Collectively these data unequivocally demonstrate that ovine prion infectivity progressively accumulated in scrapie-exposed ovine VRQ PrP transgenic *Drosophila.*

### Fly-to-fly prion propagation

We next investigated whether the prion infectivity that accumulates in VRQ *Drosophila* at first passage could be serially propagated in the same fly line. Accordingly, head homogenate from 30-day-old adult *Drosophila* (first passage flies) was used to inoculate fresh VRQ *Drosophila* (second passage flies) at the larval stage. Second passage VRQ *Drosophila* were allowed to hatch and groups of flies were euthanized at 5, 30 and 40 days of age when head homogenate was prepared for PMCA analysis. The data in the Supplementary material (S2) show, as expected, no PMCA prion seeding activity was detected in VRQ *Drosophila* that were exposed to head homogenate from first passage mock-infected VRQ *Drosophila,* or first passage scrapie- or mock infected 51D flies. However, prion seeding activity was detected in second passage VRQ *Drosophila* exposed to 30- and 40-day-old, but not 5-day-old, head homogenate from first passage scrapie-exposed VRQ *Drosophila.* Endpoint titration of the PMCA-positive second passage VRQ *Drosophila* samples indicated an increase in prion seeding activity titre in these flies between 30 and 40 days of age.

Second passage VRQ *Drosophila* head homogenate was inoculated into tg338 mice to assess prion infectivity in these samples. Mice that received 30-day-old PMCA-positive second passage VRQ *Drosophila* head homogenate showed 100% attack rate for clinical signs of mouse prion disease and an incubation period of 89 ± 4 days. The brains of inoculated mice were examined for the presence of disease-associated PrP as shown by the data in the Supplementary material (S3). PK-resistant PrP^Sc^ was evidenced by western blot of the brains of clinically affected mice and PET blot analysis of the brains of clinically affected mice revealed the typical distribution of PrP^Sc^ in PG127 scrapie-inoculated tg338 mice. No clinical signs or abnormal PrP accumulation was observed in tg338 mice inoculated with 5-day-old second passage VRQ *Drosophila* head homogenate or from VRQ *Drosophila* exposed to first passage 51D control flies. These results demonstrate that mammalian prions propagated in VRQ *Drosophila* can be serially transmitted in flies.

### Prion-induced toxic phenotype in PrP transgenic *Drosophila*

Since prion-induced toxicity occurs concomitantly with prion replication in mammalian hosts (Bueler *et al.*, 1993; Mallucci *et al.*, 2003) we next investigated whether the propagation of prions in VRQ *Drosophila* induced a toxic phenotype in these flies. To do so, we performed a negative geotaxis climbing assay (Thackray *et al.*, 2012*c*) using adult *Drosophila* previously exposed at the larval stage to ovine scrapie prions. The data in the Supplementary material (S4, together with the accompanying statistical analysis described in the Supplementary material for this figure) show that there was no difference in the climbing ability between 51D flies exposed to PG127 scrapie or control prion-free sheep brain homogenate. Strikingly, VRQ *Drosophila* developed a toxic phenotype after exposure to PG127 prions, evidenced by an accelerated decrease in locomotor ability compared to control treated flies that became progressively more severe with age. Collectively, these data show that the prion-induced toxic fly phenotype was associated with prion propagation in adult PrP transgenic *Drosophila* as evidenced by the data in Table 1 and Fig. 3.

### Prion strain properties are maintained in PrP transgenic *Drosophila*

A comparison of the incubation period and PK-resistant PrP^Sc^ molecular profile observed in tg338 mice inoculated with either a sample of the original PG127 sheep scrapie inoculum, or this material passaged in VRQ *Drosophila*, suggested that mammalian prion strain properties were unaltered following propagation in PrP transgenic *Drosophila.* To confirm this was the case we propagated three distinct ovine prion strains in PrP transgenic *Drosophila* prior to their re-isolation in tg338 mice. The panel of ovine prion strains used here have been previously characterized by bioassay in tg338 mice and are differentiated by unique incubation times and on the basis of their PK-resistant PrP^Sc^ western blot molecular profile (Thackray *et al.*, 2011, 2012*a*). We exposed ovine PrP transgenic *Drosophila* at the larval stage to each ovine scrapie prion strain. VRQ *Drosophila* were exposed to ovine prions isolated in VRQ PrP transgenic mice and ARQ *Drosophila* were exposed to ovine prions isolated in ARQ PrP transgenic mice. Collectively, the ovine PrP transgenic *Drosophila* were susceptible to all three different prion strains shown by the development of a progressive toxic phenotype in the form of an accelerated decline in performance in a negative geotaxis climbing assay (Supplementary material, S4).

**Figure 4:**
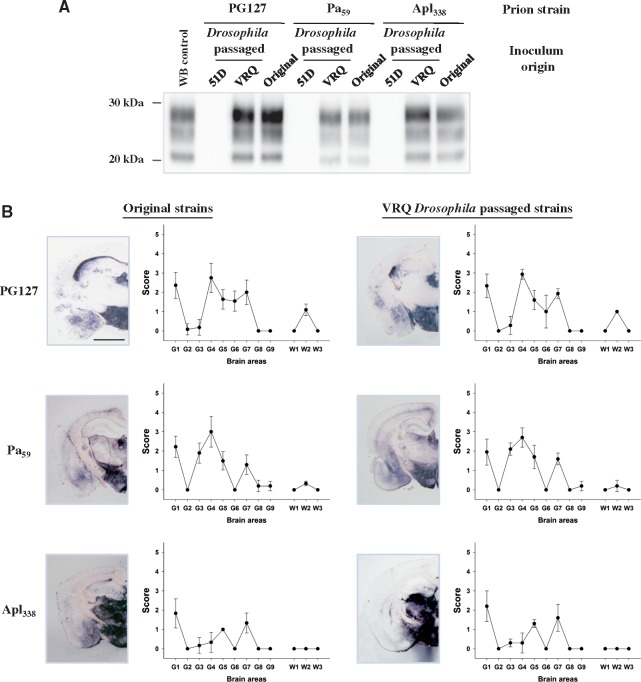
**Authentic prion replication in PrP transgenic *Drosophila.*** *Actin* × VRQ(GPI) PrP transgenic (VRQ) *Drosophila* were exposed to the PG127 and Apl_338,_ prion strains, and *Elav* × ARQ(GPI) PrP transgenic (ARQ) *Drosophila* were exposed to the Pa_59_ prion strain, at the larval stage. *Actin* × 51D and *Elav* × 51D (both referred to as 51D in appropriate graph) *Drosophila* were used as control flies where appropriate. Head homogenates were prepared from adult flies aged 30 days and inoculated into tg338 mice. Inoculated mice were euthanized when they showed clinical signs of prion infection or after 250 days for those that did not develop clinical disease. Mice were considered positive for prion disease when PK-resistant PrP27–30 was detected in brain tissue by western blot. Mouse brains were analysed for the presence of PK-resistant PrP and subjected to neuropathological assessment by PET blot and lesion profile analysis. (**A**) Western blot detection of PK-resistant PrP27–30 in the brains of tg338 mice during the isolation of PG127, Pa_59_ or Apl_338_, or prion strains (original inoculum), or these prion strains after passage in *Drosophila* (*Drosophila* passaged). Molecular mass markers in kDa are shown on the *left.* (**B**) PET blot and lesion profile analysis of tg338 mouse brains after exposure to the original mouse-derived PG127, Pa_59_ or Apl_338_, or prion strains (original inoculum), or these prion strains after passage in *Drosophila* (*Drosophila* passaged). Scale bar = 150 µm.

An aliquot of each original prion strain isolated in PrP transgenic mice and samples of these prions propagated in PrP transgenic *Drosophila* were transmitted to tg338 mice (for either one or two passages). The attack rate and incubation period observed in tg338 mice inoculated with the original prion strains were similar to those obtained following inoculation with the equivalent prion strain propagated in PrP transgenic *Drosophila* as shown in Table 2. The brains of clinically prion-diseased tg338 mice were examined for disease-associated PrP and neuropathology as shown in Fig. 4. Irrespective of the number of passages, the molecular profile of PK-resistant PrP^Sc^ observed in the brains of tg338 mice inoculated with the original ovine prions and those propagated in PrP transgenic *Drosophila* were indistinguishable (Fig. 4A). In addition, the vacuolar lesion profile in the brains of tg338 mice inoculated with the original ovine prion strain and with the equivalent prion strain propagated in PrP transgenic *Drosophila* were virtually identical (Fig. 4B). Collectively, these data demonstrate that the biological properties of distinct ovine prion strains were maintained after their propagation in ovine PrP transgenic *Drosophila.*

**Table 2:** Scrapie strains retain transmission properties after passage in PrP transgenic *Drosophila*

Scrapie strain	Original strain isolated in tg338 mice		*Drosophila*-passaged strain isolated in tg338 mice			
			First passage		Second passage	
	Attack rate	IP	Attack rate	IP	Attack rate	IP
PG127	6/6	61 ± 2	6/6	87 ± 4	6/6	59 ± 1
Pa_59_	6/6	143 ± 3	6/6	187 ± 4	6/6	142 ± 1
Apl_338_	6/6	631 ± 83	3/5	642 ± 57	NA	NA

The ovine classical scrapie prion strains PG127 and Apl_338_ (isolated in ovine VRQ tg338 mice) and Pa_59_ (isolated in ovine ARQ tg59 mice) were transmitted to tg338 ovine PrP transgenic mice. The ovine classical scrapie prion strains PG127, Pa_59_ or Apl_338_ were transmitted to tg338 ovine PrP transgenic mice. In parallel, *Actin* × VRQ(GPI) PrP transgenic (VRQ) *Drosophila* were exposed to the PG127 and Apl_338,_ prion strains, and *Elav* × ARQ(GPI) PrP transgenic (ARQ) *Drosophila* were exposed to the Pa_59_ prion strain, at the larval stage. Head homogenates were prepared from adult flies aged 30 days and serially transmitted in tg338 mice (two iterative passages) by intracerebral inoculation. Mice were euthanized when they showed clinical signs of prion infection and after 250 days for those that did not develop clinical disease. Mice were considered positive for prion disease when PK-resistant PrP27–30 was detected in brain tissue by western blot. The attack rate (number of prion positive mice/total number of mice inoculated) is reported for each treatment group. The incubation period (IP) for inoculated mice, which represents the mean time from inoculation to euthanasia for each inoculated group of animals, is reported in days ± SD. NA = data not available, still ongoing.

## Discussion

In our study presented here we have demonstrated that scrapie-exposed *Drosophila* transgenic for ovine PrP expressed pan neuronally displayed a progressive accumulation of prion seeding activity and showed the presence of PK-resistant PrP. Crucially, we also demonstrated a progressive accumulation of prion infectivity in these *Drosophila* by bioassay in tg338 mice. These features unequivocally demonstrate for the first time the replication of *bona fide* mammalian prions in a PrP transgenic invertebrate host.

The central role of PrP in prion replication has been verified by infectivity studies in mammalian hosts, principally mice, with either gene knock-out or transgenic over-expression of endogenous prion protein expression (Brandner and Jaunmuktane, 2017). These extensive studies have collectively established that mammalian prions do not replicate in hosts that fail to express PrP^C^. However, several lines of evidence have suggested that expression of PrP^C^ alone is not sufficient to confer susceptibility to prion propagation (Raeber *et al.*, 1999; Enari *et al.*, 2001; Courageot *et al.*, 2008). This has led to the suggestion that specific molecular co-factors and/or cellular machinery are crucial for prion propagation. The nature and function of co-factors in prion propagation remains undefined (Ma, 2012), although their role is highlighted by *in vitro* reconstitution experiments. To date, RNA or lipid has been shown to be required for the generation of prions with high infectious titre (Deleault *et al.*, 2003; Wang *et al.*, 2010; Deleault *et al.*, 2012). However, it is possible that other molecular components participate in the process of prion replication and the definitive list of these co-factors and their mode of action remain to be established (Castilla *et al.*, 2008). Insects are invertebrate hosts that are phylogenetically separated in evolutionary terms from mammals by millions of years. We have demonstrated here that *Drosophila*, a normally PrP-null insect host, rendered transgenic solely for mammalian PrP, are permissive for the faithful replication of a panel of three mammalian prion strains. This provides a cogent argument that the nature of the molecular and cellular factors that facilitate prion replication do not result from a co-evolutionary selection process between the infectious agent and their natural hosts.

An intriguing feature in mammalian prion biology is the existence of prion strains. These are identified as different prion isolates from individuals of the same species that produce distinct disease phenotypes upon serial passage in isogenic hosts (Bruce and Dickinson, 1987). Glycosylation is an important factor in the determination and maintenance of conformation, function and interactions of glycoproteins (O’Connor and Imperiali, 1996). Mammalian PrP expressed in the natural host has two *N*-linked carbohydrate moieties attached to asparagine residues located in the C-terminal domain of the protein (Endo *et al.*, 1989). Transmission experiments carried out in mice transgenic for PrP devoid of either one or both *N*-linked carbohydrate moieties, as a result of changes to the prion protein primary structure, indicated an alteration in the biological properties of some of the prion strains tested (Cancellotti *et al.*, 2013). These results supported the view that *N*-linked glycosylation of PrP^C^ could participate in the transfer and/or maintenance of the prion strain-specific information. Mammalian *N*-linked glycoproteins, including those attached to PrP^C^ expressed in the natural host, typically comprise complex glycans that consist of *N*-acetylglucosamine (GlcNAc), mannose, galactose, and terminal sialic acid residues (Endo *et al.*, 1989). In contrast, the majority of neurons in the *Drosophila* brain synthesize *N*-linked glycans with core structures similar or identical to those produced by all eukaryotes but fail to acquire complex carbohydrate structures (März *et al.*, 1995). Our study here demonstrates that the biological properties of distinct ovine prion strains were maintained after propagation in PrP transgenic *Drosophila.* Therefore, differences in *N*-linked glycosylation moieties between mammalian and insect species had no apparent impact on the biological properties of the different prion strains that we propagated in PrP transgenic *Drosophila.*

We have shown that VRQ *Drosophila* exposed to scrapie prions at the larval stage show an accelerated decline in locomotor ability at adulthood (Thackray *et al.*, 2012*b*, 2014, 2016). The severity of this prion-induced toxic fly phenotype increased as the flies aged and correlates with the accumulation of prion seeding activity and infectivity in PrP transgenic *Drosophila.* In mammalian species clinical signs in prion affected individuals arise as a consequence of neurodegenerative processes initiated by the conversion of PrP^C^ to PrP^Sc^. Only cells that express PrP^C^ are susceptible to prion-induced toxicity and formation of the toxic moiety occurs concomitantly with prion replication (Bueler *et al.*, 1993; Mallucci *et al.*, 2003). Despite significant advances (Moreno *et al.*, 2012), the mechanisms responsible for neurodegeneration in prion disease and the role of PrP^C^ and PrP^Sc^ remain incompletely defined. Unravelling events that are directly triggered by prion protein conversion from those arising as a consequence of the perturbation of the CNS micro-environment is extremely challenging. PrP was expressed pan neuronally in the VRQ PrP transgenic fly line used to assess prion seeding activity and prion infectivity in the scrapie-infected *Drosophila* we have used here. We have previously shown that a prion-induced toxic phenotype occurs in *Drosophila* transgenic for pan neuronal expression of PrP (Thackray *et al.*, 2014, 2016). Consequently, we consider that the prion-induced decline of the locomotor activity observed in PrP transgenic *Drosophila* reflects a neurotoxic phenotype that arises as a consequence of prion replication. This supports the view that PrP transgenic *Drosophila* can serve as a tractable system to probe for genetic modifiers of prion-induced neurodegeneration. *Drosophila* are increasingly used as a model system in the study of mammalian neurodegenerative disease including the pathobiology of Alzheimer’s disease, Parkinson’s disease and tauopathies (Lu and Vogel, 2009). This arises because the brains of *Drosophila* and mammalian species are composed of similar components (i.e. neurons and neuronal circuitry), and the nature of ion channels, neurotransmitters and synaptic proteins are highly conserved between mammals and the fly (Lessing and Bonini, 2009). Our use of PrP transgenic *Drosophila* to recapitulate mammalian prion infection provides an unprecedented opportunity to unravel the mechanisms of prion-induced neurotoxicity in a new animal system. *Drosophila* have already proved to be an important animal model to analyse the genetics of mammalian neurodegeneration. For example, resultant behavioural defects in *Drosophila* that arise through the deletion of a fly homologue of a human neurodegenerative gene can be compensated for by a functional human homologous gene (Luo *et al.*, 1992). In addition, mutagenesis screens in *Drosophila* have led to the isolation of flies that show late-onset progressive degeneration of the adult nervous system that resembles human diseases (Min and Benzer, 1999).

Prion bioassays, which are generally carried out in conventional or transgenic mice, or primates or small ruminants, continue to provide a central role in the study of mammalian prion diseases. However, because of ethical and economic reasons, such as the cost of maintaining the vertebrate species during long bioassays, there is increasing pressure to limit their use and adopt alternative approaches to assess mammalian prion infectivity. Over the last decade, *in vivo* prion biology studies have been supplemented by *in vitro* techniques such as PMCA (Saa *et al.*, 2005) and quaking-induced conversion (QuIC) (Atarashi *et al.*, 2007). Both of these methodologies have been used for the detection of prion seeding activity, a biochemically and biologically relevant surrogate marker of prion infectivity. In this context, PMCA and QuIC provide rapid and sensitive quantitative detection of prion-infected samples. However, *in vitro* amplification methods remain refractory to many questions and experimental needs that require to be addressed in prion research. These methods are also obviously not adapted for investigating prion-induced neurodegeneration. *Drosophila* have several important positive experimental advantages as an animal model to study neurodegenerative disease. Large numbers of flies can be produced in a short time, which correlated with their relatively short life span and simple genetics, allow rapid, statistically robust data collection. The ability of *Drosophila* to faithfully propagate mammalian prion strains has multiple potential applications. In relation to prion diagnostics, our *Drosophila*-based prion bioassay has already been shown to be capable of the sensitive detection of prion infectivity in brain tissue (Thackray *et al.*, 2012*b*, 2016) and blood (Thackray *et al.*, 2016). PrP transgenic *Drosophila* could also help to address the need for new animal models that allow a relevant, rapid, robust and reasonably high throughput screening of therapeutic compounds against prion disease.

In our studies presented here we have demonstrated that core features of transmissible mammalian prion disease can be recapitulated in PrP transgenic *Drosophila.* This opens a new era for the investigation of prion-induced neurotoxicity and prion replication mechanisms in a genetically well-defined tractable animal system. This new invertebrate model of mammalian prion disease will provide the opportunity to exploit the power of genetics in the fly to identify potential genetic modifiers of prion-induced neurotoxicity. Such genetic modifiers may serve as candidate diagnostic markers or therapeutic targets of human prion disease and prion-like diseases. In addition, the ease of transgenesis in *Drosophila* will allow the development of fly lines that express different species forms of PrP, such as bovine, cervid and human PrP to bioassay prion infectivity from these different mammalian hosts. For appropriate biosecurity, these future studies will require the BSE, chronic wasting disease (CWD) or CJD-inoculated *Drosophila* to be maintained at containment level 3 for prion research, an enhanced level of safety above the containment level 2 used for the sheep scrapie studies described here. In this manner, PrP transgenic *Drosophila* can be used to begin to address important questions on the pathogenic potential of known and possible zoonotic prions.

## Funding

This work was supported in part by funds from the NC3Rs Project (Grant NC/K000462/1).

## Supplementary Material

Supplementary Data

## Abbreviations

ARQ: A^136^R^154^Q^171^ ovine PrP
GPI: glycosylphosphatidyl inositol
PMCA: protein misfolding cyclic amplification
PrP^C^: cellular prion related protein
PrP^Sc^: disease-associated conformers of prion related protein
VRQ: V^136^R^154^Q^171^ ovine PrP
