# Supplementary material for: Mammalian prion propagation in PrP transgenic Drosophila
Source: Brain. 2018 Jul 8;141(9):2700–10. doi: 10.1093/brain/awy183 (PMC6113635; doi:10.1093/brain/awy183)

Supplementary Materials: Mammalian prion propagation in PrP transgenic *Drosophila*

Introduction

We have successfully demonstrated that transmissible prions replicate in scrapie-exposed ovine PrP transgenic *Drosophila* (see main paper). In order to establish the robustness of our observations we performed repeat fly-to-mouse transmission experiments. We also investigated whether the prions that replicate in scrapie-exposed *Drosophila* were serially transmissible in the same fly line. In addition, we investigated whether prion replication in the fly was associated with prion-induced toxic phenotype as assessed by a negative geotaxis climbing assay.

Results and Discussion

The data in Supplementary S1 show the attack rate (number of prion positive mice/total number of mice inoculated) and incubation period (IP) for repeat fly-mouse prion transmission experiments were similar to those observed in the original experiment shown in Table 1 of the main text. Collectively these data unequivocally demonstrate that ovine prion infectivity progressively accumulated in scrapie-exposed ovine VRQ PrP transgenic *Drosophila*.

The data in Supplementary S2 show that the prion seeding activity that accumulates in scrapie-exposed VRQ *Drosophila* at first passage could be serially propagated in the same fly line. No PMCA prion seeding activity was detected in VRQ *Drosophila* exposed to head homogenate from first passage mock-infected VRQ *Drosophila,* or first passage scrapie- or mock infected 51D flies. Prion seeding activity was detected in second passage VRQ *Drosophila* exposed to 30 and 40 day old, but not 5 day old, head homogenate from first passage scrapie-exposed VRQ *Drosophila*. Endpoint titration of the PMCA-positive second passage VRQ *Drosophila* samples indicated an increase in prion seeding activity titre in these flies between 30 and 40 days of age. These data demonstrate fly-to-fly prion propagation.

Second passage VRQ *Drosophila* head homogenate was inoculated into tg338 mice in order to assess the level of prion infectivity in these samples. Mice that received 30

day old PMCA-positive second passage VRQ *Drosophila* head homogenate showed 100% attack rate for clinical signs of mouse prion disease and an incubation period of 89±4 days. The brains of inoculated mice were examined for the presence of disease-associated PrP as shown by Supplementary S3. PK-resistant PrP^{Sc} was evidenced by western blot in the brains of clinically affected mice (Supplementary S3A). PET blot analysis of the brains of clinically affected mice showed the typical distribution of PrP^{Sc} in PG127 scrapie-inoculated tg338 mice (Supplementary S3B). No clinical signs or abnormal PrP accumulation was observed in tg338 mice inoculated with 5 day old positive second passage VRQ *Drosophila* head homogenate or from VRQ *Drosophila* exposed to first passage 51D control flies. These results demonstrate that mammalian prions propagated in VRQ *Drosophila* can be serially transmitted in flies.

We investigated whether prion replication in scrapie-exposed PrP transgenic *Drosophila* induced a toxic phenotype in these flies. To do so, PrP transgenic *Drosophila* were exposed at the larval stage to prion-infected or prion-free tg338 or tg59 mouse brain homogenate. After hatching, the locomotor ability of prion-exposed and control *Drosophila*, expressed as a performance index, was assessed by a negative geotaxis climbing assay (Thackray et al., 2012c). The data in Supplementary S4 show the locomotor ability of adult *Drosophila* after exposure to ovine prions at the larval stage. Supplementary S4A shows that VRQ *Drosophila* displayed an accelerated decline in locomotor activity after exposure to both Apl_{338} and PG127 prion strains, which was significantly different to the response seen by that of similar flies exposed to scrapie-free tg338 mouse brain homogenate (p=0.0001, and p=0.0008, respectively, assessed over days 1 - 40). The data in Supplementary S4B show that the locomotor ability of ARQ *Drosophila* after exposure to Pa_{59} prions displayed a decline in locomotor activity that was significantly different to the response seen by that of similar flies exposed to scrapie-free mouse brain homogenate (p=0.0006, assessed over days 1 - 40). In contrast to these data, Supplementary S4C shows that control 51D flies displayed no difference in their locomotor ability after exposure to prion-infected or scrapie-free mouse brain homogenate (p>0.05 in all cases, except Apl_{338} p=0.0227, assessed over days 1 - 40). Collectively, these data show that PrP transgenic *Drosophila* are sensitive to prion-induced toxicity mediated by different prion strains.

Supplementary Data Legends

Supplementary S1: Reproducible fly-to-mouse transmission of prions from scrapie-exposed PrP transgenic *Drosophila*

Elav x VRQ(GPI) PrP transgenic (VRQ) and *Elav* x 51D (51D) *Drosophila* were exposed at the larval stage to PG127 scrapie-infected or prion-free control sheep brain material. At 30 days post hatching, head homogenate was prepared from harvested flies and inoculated into tg338 mice. Mice were euthanized when they showed clinical signs of prion infection and after 250 or 670 days post-inoculation for those that did not develop clinical disease. Mice were considered positive for prion disease when PK-resistant PrP27–30 was detected in brain tissue by western blot. The attack rate (number of prion positive mice/total number of mice inoculated) is reported for each treatment group. The incubation period (IP) for inoculated mice, which represents the average time from inoculation to euthanasia for each inoculated group of animals, is reported in days ± SD.

Supplementary S2. Serial transmission of prion seeding activity in scrapie-exposed PrP transgenic *Drosophila*

PG127 scrapie-infected or control prion-free sheep material was passaged in *Elav* x VRQ(GPI) PrP transgenic (VRQ) and *Elav* x 51D (51D) *Drosophila* (primary passage) and subsequently in *Elav* x VRQ(GPI) PrP transgenic (VRQ) flies (secondary passage). At various times after hatching, head homogenate was prepared from harvested flies and used as seed in PMCA reactions. **(A)** End-point titration of PMCA prion seeding activity in head homogenate from control or PG127-exposed *Drosophila*; (**B**) western blot analysis of PK-resistant PrP27–30 in PMCA reaction products seeded with *Drosophila* head homogenate. Molecular mass markers in kDa are shown on the left.

Supplementary S3. Serial transmission of prion infectivity in scrapie-exposed PrP transgenic *Drosophila*

PG127 scrapie-infected or control prion-free sheep material was passaged in *Elav* x VRQ(GPI) PrP transgenic (VRQ) and *Elav* x 51D (51D) *Drosophila* (primary passage) and subsequently in *Elav* x VRQ(GPI) PrP transgenic (VRQ) flies (secondary

passage). Head homogenate from second passage *Elav* x VRQ(GPI) PrP transgenic (VRQ) *Drosophila* was inoculated into tg338 mice. Inoculated mice were euthanized when they showed clinical signs of prion infection or after 250 days for those that did not develop clinical disease. Mice were considered positive for prion disease when PK-resistant PrP27–30 was detected in brain tissue by western blot. (**A**) Western blot detection of PK-resistant PrP27–30 in the brains of tg338 mice with clinical prion disease. Molecular mass markers in kDa are shown on the right; **(B)** PET blot analysis of the brains from tg338 mice inoculated with 30 day old 2nd passage *Elav* x VRQ(GPI) PrP transgenic (VRQ) *Drosophila*. Scale bar represents 150 µm.

Supplementary S4. Prion-induced toxicity in scrapie-exposed PrP transgenic *Drosophila*

Drosophila were assessed for their locomotor ability by a negative geotaxis climbing assay following exposure at the larval stage to PG127 (red line), Apl_{338} (light blue line); Pa_{59} (green line) prion-infected, or prion-free tg338 (grey line) or tg59 (black line) ovine PrP transgenic mouse brain homogenate. The data shown are linear regression plots of the mean performance index ± SD for three groups of flies per time point calculated as described in the Materials and Methods. *p* values assessed over days 1 – 40 of the climbing assay are shown on individual graphs. (**A)** *Actin* x VRQ(GPI) PrP transgenic (VRQ) *Drosophila*; **(B)** *Elav* x ARQ(GPI) PrP transgenic (ARQ) *Drosophila*; **(C)** *Actin* x 51D (green and black lines) and *Elav* x 51D (blue, red and grey lines).

Supplementary Data S1: Reproducible fly-to-mouse transmission of prions from scrapie-exposed PrP transgenic *Drosophila*

Experiment	Fly line	Inoculum	Transmission in tg338 mice: Attack Rate	Transmission in tg338 mice: IP
1	51D	Control	0/6	>670
		PG127	0/6	>670
	VRQ	Control	0/6	>250
		PG127	6/6	87 ± 4
2	51D	Control	0/6	>670
		PG127	0/6	>250
	VRQ	Control	0/6	>250
		PG127	6/6	87 ± 1
3	51D	Control	0/6	>250
		PG127	0/6	>250
	VRQ	Control	0/6	>250
		PG127	6/6	87 ± 2

Supplementary Data S2. Serial transmission of prion seeding activity in scrapie-exposed PrP transgenic *Drosophila*

(A)

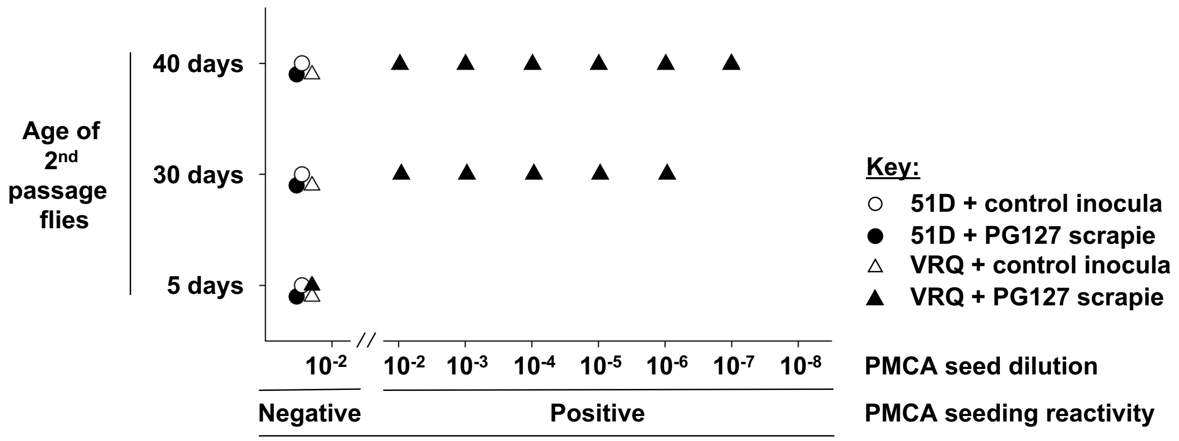

(B)

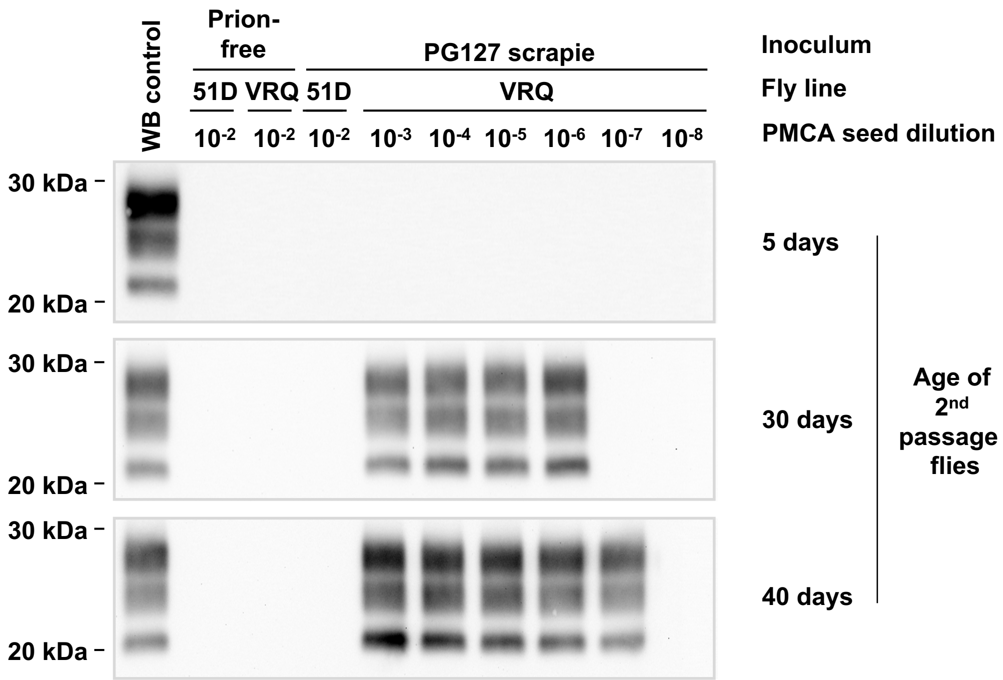

Supplementary Data S3. Serial transmission of prion infectivity in scrapie-exposed PrP transgenic *Drosophila*

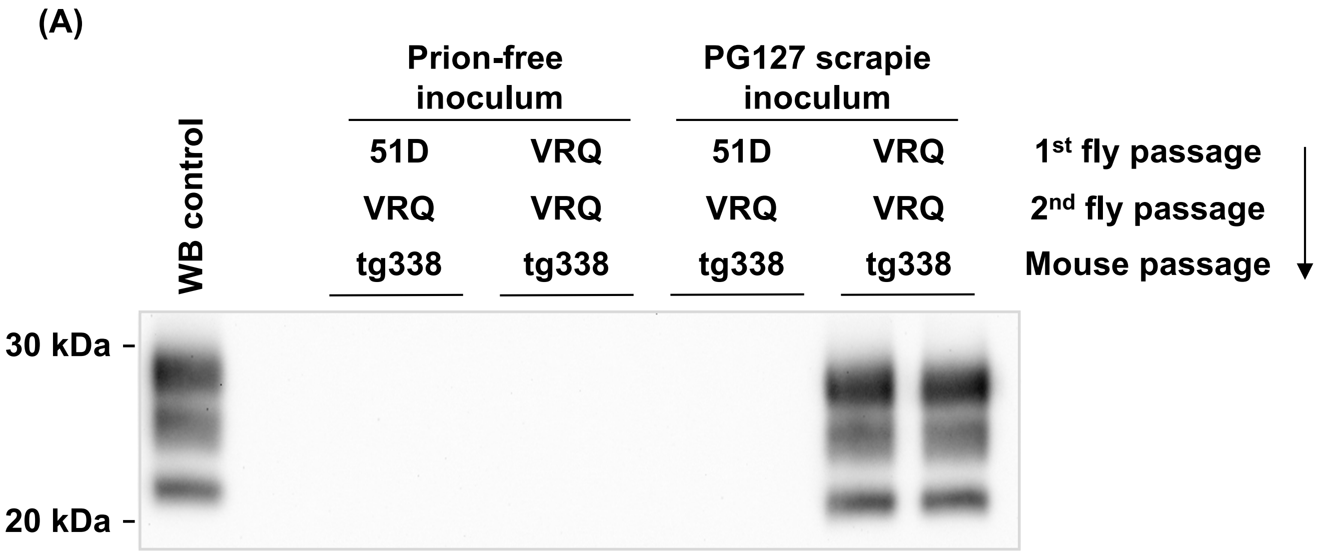

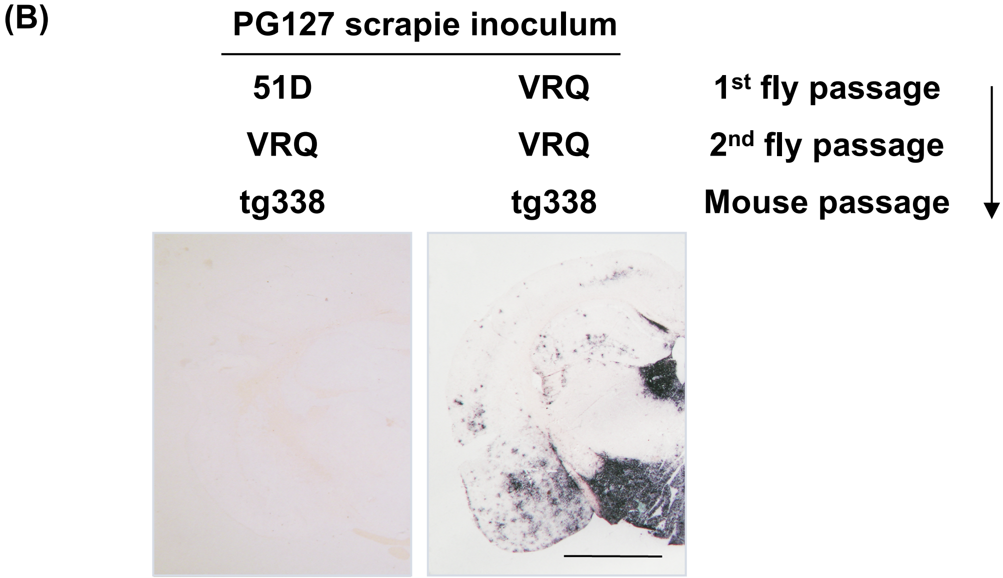

Supplementary Data S4: Prion-induced toxicity in scrapie-exposed PrP transgenic *Drosophila*

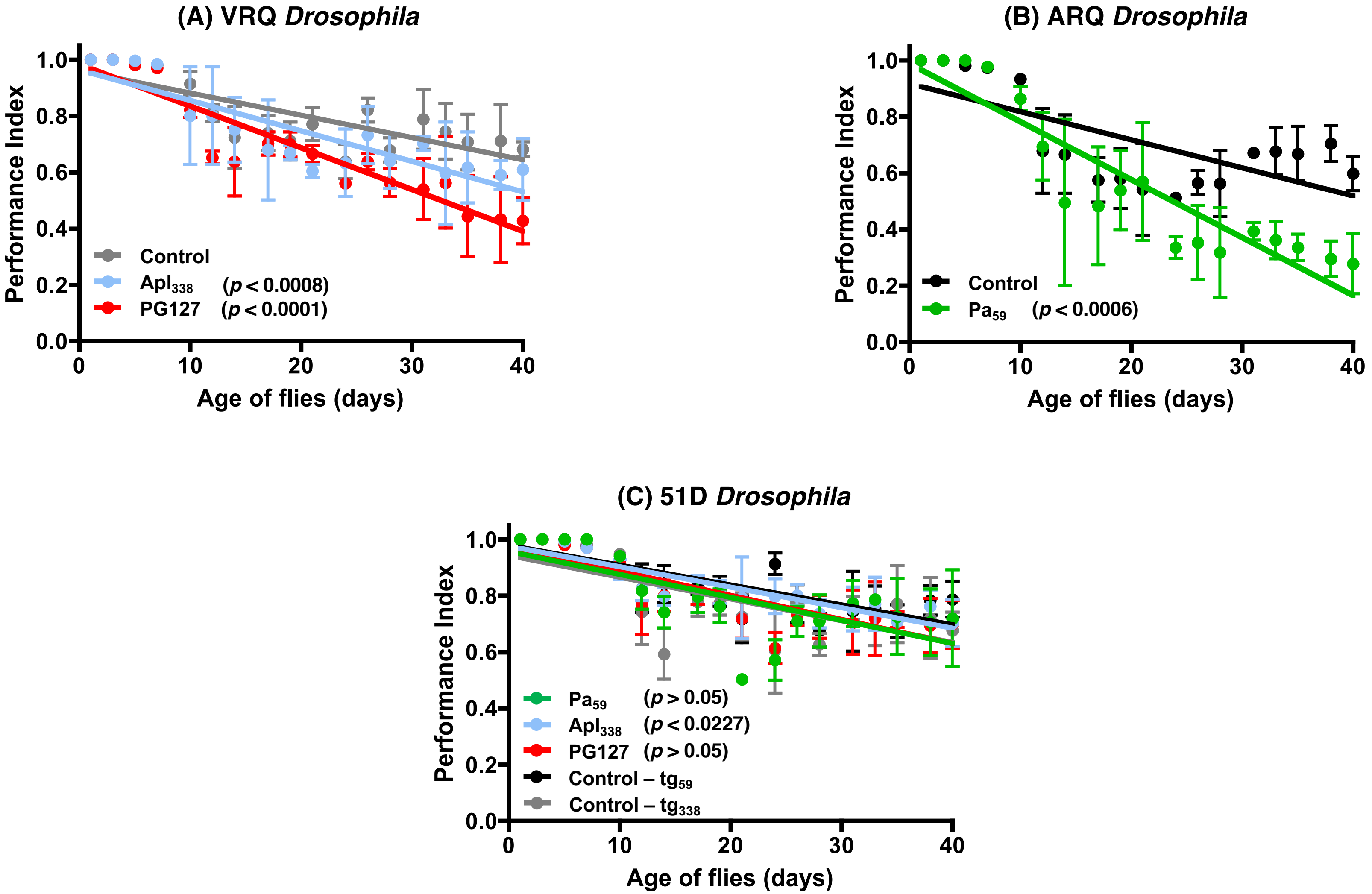

Supplement: Supplementary Data [file awy183_supp.pdf]
